# A systematic optimization of styrene biosynthesis in *Escherichia coli* BL21(DE3)

**DOI:** 10.1186/s13068-018-1017-z

**Published:** 2018-01-25

**Authors:** Changqing Liu, Xiao Men, Hailin Chen, Meijie Li, Zhaorui Ding, Guoqiang Chen, Fan Wang, Haobao Liu, Qian Wang, Youshuang Zhu, Haibo Zhang, Mo Xian

**Affiliations:** 1 CAS Key Laboratory of Biobased Materials, Qingdao Institute of Bioenergy and Bioprocess Technology, Chinese Academy of Sciences, No.189 Songling Road, Laoshan District, Qingdao, 266101 China; 2 University of Chinese Academy of Sciences, Beijing, China; 3 Key Laboratory for Tobacco, Gene Resources’ Tobacco Research Institute, Chinese Academy of Agricultural Sciences, Qingdao, 266101 People’s Republic of China; 4 School of Biological Science, Jining Medical University, Jining, 272067 People’s Republic of China

**Keywords:** Styrene, l-phenylalanine, Phenylalanine ammonia lyase, *aroF*, *pheA*, *tktA*, *ppsA*, In situ product removal, Isopropyl myristate, *Escherichia coli*

## Abstract

**Background:**

Styrene is a versatile commodity petrochemical used as a monomer building-block for the synthesis of many useful polymers. Although achievements have been made on styrene biosynthesis in microorganisms, several bottleneck problems limit factors for further improvement in styrene production.

**Results:**

A two-step styrene biosynthesis pathway was developed and introduced into *Escherichia coli* BL21(DE3). Systematic optimization of styrene biosynthesis, such as enzyme screening, codon and plasmid optimization, metabolic flow balance, and in situ fermentation was performed. Candidate isoenzymes of the rate-limiting enzyme phenylalanine ammonia lyase (PAL) were screened from *Arabidopsis thaliana* (AtPAL2), *Fagopyrum tataricum* (FtPAL), *Petroselinum crispum* (PcPAL), and *Artemisia annua* (AaPAL). After codon optimization, AtPAL2 was found to be the most effective one, and the engineered strain was able to produce 55 mg/L styrene. Subsequently, plasmid optimization was performed, which improved styrene production to 103 mg/L. In addition, two upstream shikimate pathway genes, *aroF* and *pheA*, were overexpressed in the engineered strain, which resulted in styrene production of 210 mg/L. Subsequently, combined overexpression of *tktA* and *ppsA* increased styrene production to 275 mg/L. Finally, in situ product removal was used to ease the burden of end-product toxicity. By using isopropyl myristate as a solvent, styrene production reached a final titer of 350 mg/L after 48 h of shake-flask fermentation, representing a 636% improvement, which compared with that achieved in the original strain.

**Conclusions:**

This present study achieved the highest titer of de novo production of styrene in *E. coli* at shake-flask fermentation level. These results obtained provided new insights for the development of microbial production of styrene in a sustainable and environment friendly manner.

**Electronic supplementary material:**

The online version of this article (10.1186/s13068-018-1017-z) contains supplementary material, which is available to authorized users.

## Background

Biorenewable fuels and chemicals have been receiving more and more attention owing to the increasing depletion of fossil fuel resources and concerns about sustainable development of conventional petrochemical industry. Currently, a number of natural and artificial biochemicals, such as succinic acid [1], lactic acid [2], isoprene [3], α-pinene [4], *n*-butanol [5], 2-phenylethanol [6], sabinene [7], as well as numerous fatty acids [8], and their derivatives [9] can be synthesized from renewable substrates such as glucose by microbes. Along with the development of metabolic engineering and the discovery of new metabolic routes, compounds that were previously unobtainable are becoming available through biosynthesis in microbes.

**Fig. 1:**
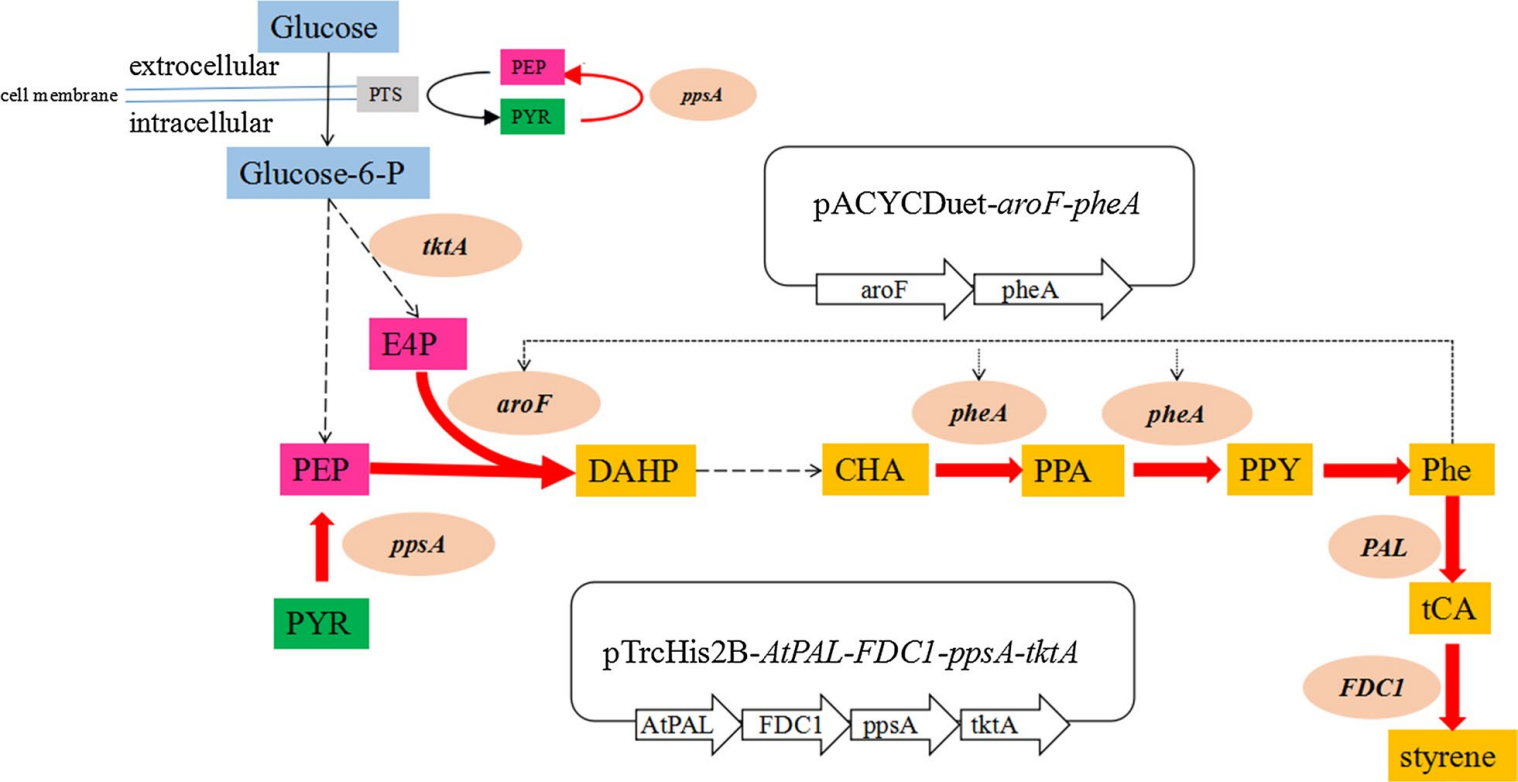
Genes related to styrene biosynthesis in *E. coli*. Metabolites abbreviations: *PTS* phosphotransferase system, *Glucose-6-P* glucose 6-phosphate, *E4P* erythrose 4-phosphate, *PEP* phosphoenolpyruvate, *DAHP* 3-deoxy-d-arabino-heptulosonate-7-phosphate, *PYR* pyruvate, *CHA* chorismate, PPA prephenate, *PPY* phenylpyruvate, *PHE* phenylalanine. Genes and enzymes: *aroF*, DAHP synthetase feedback inhibited by Tyr; *pheA* fused chorismate mutase and prephenate dehydratase, *tktA* transketolase, *ppsA* phosphoenolpyruvate synthase, *PAL* phenylalanine ammonia lyase, *FDC1* ferulic acid decarboxylase

Styrene is a versatile commodity chemical, with an annual global consumption of approximates 25 million metric tons and occupying an approximately 30 billion USD market [10]. Currently, almost all commercially available styrene is derived from the increasing depletion of fossil fuel resources that are rapidly being depleted. Moreover the demand for sustainable resources has impelled the researchers to develop biosynthetic routes for the production of styrene from renewable substrates. Recently, a synthetic biological pathway was employed for production of styrene directly from glucose [11]. The two-step heterologous pathway was designed as follows: l-phenylalanine (l-Phe) was converted to trans-cinnamic acid (ferulic acid, tCA), catalyzed by phenylalanine ammonia lyase (PAL, EC 4.3.1.24). Subsequently, tCA was converted to styrene, catalyzed by ferulic acid decarboxylase (FDC1, EC 4.1.1.102, a phenyl acrylate decarboxylase) (Fig. 1). This pathway was first expressed in *Escherichia coli* NST74, which was previously developed for l-Phe production and the resulting strain was able to produce 260 mg/L styrene from 15 g/L glucose, with a styrene toxicity threshold of 300 mg/L [11]. However, low PAL activity acted as the flux limiting condition in the engineered styrene biosynthesis pathway because enough l-Phe titers were detected, while tCA titers were observed to remain low throughout, indicating that almost all of the synthesized tCA could be quickly converted to styrene by overexpressed* FDC1* [11]. Moreover, although addition of exogenous l-Phe significantly improved the net production of styrene, this approach towards improving styrene biosynthesis is not sustainable, and endogenous l-Phe production must be enhanced in the host platform [11]. Besides, styrene toxicity was a limiting factor, which must be addressed if bio-styrene becomes an alternative to traditional chemical processes [11].

PAL is the first enzyme that leads the l-Phe metabolism into styrene biosynthesis, and its activity has significant effects on styrene yield. To enhance styrene production, one of the most common strategies is to search for PAL with higher activity by screening new isoenzymes from different sources. Nielsen et al. examined four PAL isoenzymes, from *Arabidopsis thaliana* (AtPAL1 and AtPAL2), *Nostoc puntiformes* (NpPAL), and *Anabaena variabilis* (AvPAL), respectively, for their suitability to produce tCA in *E. coli*, and found that AtPAL2 had the highest enzyme activity among the four isoenzymes investigated [11]. Moreover, although directed evolution and random mutagenesis have been widely used to enhance enzymatic activity, PAL with higher activity has not yet been reported [12].

Numerous methods to increase endogenous l-Phe have been developed [13–18]. It has been demonstrated that l-Phe exhibits feedback inhibition on some of its key biosynthesis pathway genes, such as *aroF* and *pheA*, encoding 3-deoxy-d-arabino-heptulosonate-7-phosphate synthetase (DAHPS), and chorismate mutase-prephenate dehydratase (CM-PDT), respectively. Overexpression or deregulation of these key genes could remarkably enhance the biosynthesis of the downstream aromatic compounds. In a previous study, zhou et al. co-expressed a feedback-resistant *aroF*^fbr^ and a wild-type *pheA*^wt^ in an l-Tyr auxotrophic *E. coli* strain WSH-Z06, and increased l-Phe production to 35.38 g/L, which was 2.81-fold higher than that achieved in the original strain [19].

Another essential strategy to improve l-Phe production is to enhance the availability of intracellular phosphoenolpyruvate (PEP) and erythrose 4-phosphate (E4P), which are two import precursors involved in l-Phe biosynthesis, from the central metabolic pathway. Overexpression of the key central metabolic pathway genes *ppsA* and *tktA* genes is known to increase the availability of PEP and E4P, respectively, and introduction of these genes has been reported to increase aromatic compounds production in engineered *E. coli* strains [20, 21]. Many biotechnological processes are limited owing to product cytotoxicity or formation of toxic by-products. To avoid these drawbacks, in situ product removal (ISPR) techniques have been developed. This strategy has been demonstrated to be effective in improving the bio-production of some aromatic compounds, such as l-Phe and 2-phenylethanol [22–25].

In the present study, a systematic optimization of styrene biosynthesis in *E. coli* BL21(DE3) was performed. To obtain PAL with higher activity, candidate isoenzymes from different sources were screened and their encoding genes were optimized based on the preferred codon usage of *E. coli*. Then, the optimized pathway genes were cloned into different plasmids to select for the suitable plasmid that was effective for enzyme expression and styrene production. Subsequently, four upstream pathway genes *aroF*, *pheA*, *tktA,* and *ppsA* were overexpressed to increase the carbon flow to l-Phe. Finally, to alleviate the toxic effect of endogenous styrene on host cells, ISPR with different solvents on engineered *E. coli* cultures was investigated.

## Results and discussion

### Screening PAL isoenzymes and codon optimization

**Fig. 2:**
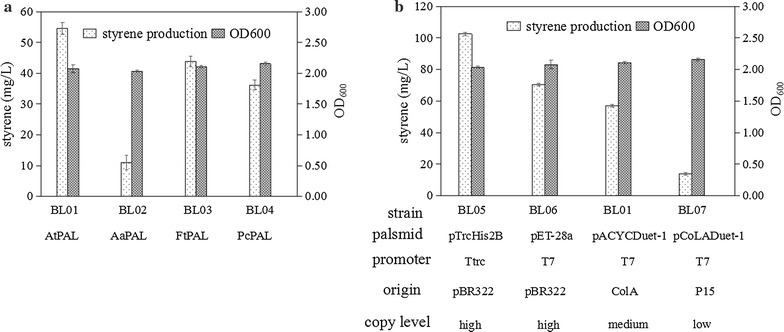
Effect of *PALs* and different plasmids on styrene production. **a** Styrene production with the expression of different *PAL* candidate genes from *P. crispum* (PcPAL), *F. tataricum* (FcPAL), *A. annua* (AaPAL), and *A. thaliana*(AtPAL) in recombinant *E. coli* BL21(DE3). **b** Styrene production in recombinant *E. coli* BL21(DE3) harboring different plasmids (pTrcHis2B, pET-28a, pACYCDuet-1, and pColADuet-1) in recombinant *E. coli* BL21(DE3). Error bars represent one standard deviation from triplicate experiments

In a previous study, Nielsen et al. confirmed that PAL was the rate-limiting enzyme in the styrene biosynthesis because the tCA titers remained low throughout [11]. In this study, to screen for a more efficient PAL, three candidate isoenzymes from *Petroselinum crispum* (PcPAL), *Fagopyrum tataricum* (FcPAL), and *Artemisia annua* (AaPAL), were screened with AtPAL2 as a control. It has been reported that recombinant PcPAL exhibits activity towards its natural substrate l-Phe, with a *Km* value of 116 ± 4 and *Kcat* value of 1 ± 0.05, and might have better catalytic properties [26]. Both FtPAL and AaPAL come from medicinal and nutrient-rich plants with high levels of flavonoids, and recombinant FtPAL protein has been found to be specific to l-Phe, with an activity of up to 35.7 IU/g [27, 28]. In the present study, to improve expression level, codon-optimized versions of all the above-mentioned genes were synthesized. *FDC1* from *Saccharomyces cerevisiae* and optimized *PALs* were cloned into the pACYCDuet-1 vector and introduced into *E. coli* BL21(DE3), and the obtained transformants were inoculated into M9 medium for 24 h. As shown in Fig. 2a, the expression of *FDC1* with *AtPAL2* led to production of 55 mg/L styrene, which was higher than that achieved with *FtPAL* (44 mg/L) or *PcPAL* (36 mg/L), respectively, whereas the strains containing *AaPAL* rarely produced styrene. Although no enzyme with higher activity was detected, both FtPAL and PcPAL were confirmed to be specific to L-Phe because gas chromatography–mass spectrometry (GC–MS) did not detect 4-Vinylphenol in the fermentation products (Additional file 1: Figure S1). Based on these results, the expression of *AtPAL2* combined with *FDC1* was used for the production of styrene in subsequent experiments.

### Selection of a suitable plasmid for increasing styrene production

Production of recombinant proteins in *E. coli* cells is affected by the number of plasmids, as well as their structural and segregational stability, which have essential impacts on productivity [29]. To achieve a high styrene producing capability, a two-step pathway was incorporated into four plasmids with different copy numbers and different promoters to assess any effect on styrene production. All the strains produced styrene from an initial glucose concentration of 15 g/L under aerobic conditions in 600-mL flasks. As shown in Fig. 2b, the strains with high-copy-number plasmids achieved higher styrene titers. *E. coli* BL06 harboring high-copy-number plasmid pET-28a-*AtPAL2*-*FDC1* produced 70 mg/L styrene, which had a 123 and 507% improvement compared with that achieved in *E. coli* BL01 harboring the medium-copy-number plasmid pACYCDuet-*AtPAL2*-*FDC1* and *E. coli* BL07 harboring the low-copy-number plasmid pColADuet-*AtPAL2*-*FDC1*, respectively. In contrast, plasmid copy number had little effect on cell growth during the process of styrene fermentation, with OD_600_ of all strains were around 2.0 (Fig. 2b). In addition to the effect of plasmid copy number, the promoter also had a significant influence on styrene production. *E. coli* BL05 harboring trc promoter plasmid pTrcHis2B-*AtPAL2*-*FDC1* produced 103 mg/L styrene under the same conditions, which was a 146% higher than that produced by *E. coli* BL06 harboring T7 promoter plasmid pET-28a-*AtPAL2*-*FDC1*. Based on these results, *E. coli* BL05 harboring plasmid pTrcHis2B-*AtPAL2*-*FDC1* was chosen as the parent strain for further styrene production optimization.

### Effect of co-expression of upstream genes *aroF* and *pheA* on styrene production

**Fig. 3:**
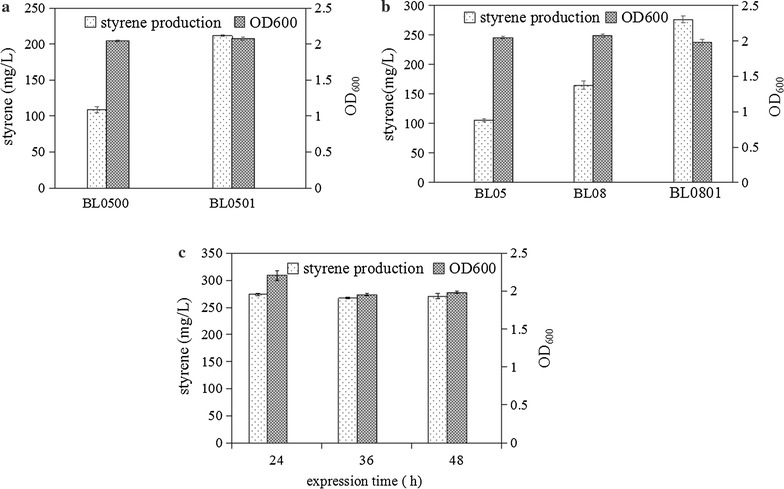
Effects of overexpression of key upstream genes on styrene production. **a** Effect of overexpression of *aroF* and *pheA* genes on styrene production. *E. coli* BL0501 and BL0500 cells were cultivated and their cell growth (OD600) and styrene production titers were compared. **b** Effect of overexpression of *aroF*, *pheA*, *ppsA*, and *tktA* genes on styrene production. *E. coli* BL0801, BL08, and BL0500 cells were cultivated and their cell growth (OD600) and styrene production titers were compared. **c** Optimization of induction length. *E. coli* BL0801 cells were induced for 24, 36, and 48 h and their cell growth (OD600) and styrene production titers were compared. Error bars represent one standard deviation from triplicate experiments

Aromatic amino acids such as l-Phe are naturally produced mainly from the shikimate pathway. The first rate-limiting step in this pathway is the condensation reaction between PEP and E4P to form 3-deoxy-d-arabino-heptulosonate 7-phosphate (DAHP). This reaction is catalyzed by three DAHPS isoenzymes encoded by three genes *aroF*, *aroG*, and *aroH*, respectively. The second rate-limiting step is the conversion of chorismate into phenylpyruvate, via prephenate, catalyzed by CM-PDT, which is encoded by *pheA*. In a previous study, Backman et al. utilized a genetically modified *E. coli* strain with *pheA*^fbr^ and *aroF*^fbr^ genes to improve the metabolic flux towards l-Phe biosynthesis and achieved 50 g/L l-Phe with a yield of 0.25 (mol l-Phe/mol glucose) [30], which was the highest l-Phe production reported thus far. In our study, to enhance upstream pathway flux, *aroF* and *pheA*, were overexpressed in *E. coli* BL05 and the resultant strain *E. coli* BL0501 was evaluated for its ability to produce styrene in shake flask. After 24 h of cultivation, the styrene titers produced by *E. coli* BL0501 reached 210 mg/L, while the control strain *E. coli* BL0500 produced 100 mg/L styrene (Fig. 3a). These results confirmed that *aroF* and *pheA* genes are the key factors determining the biosynthesis of endogenous l-Phe and co-expression of CM-PDT and DAHPS could significantly improve l-Phe and l-Phe derivatives produced by *E. coli*, similar to that reported in previous studies [31, 32].

### Effect of co-expression of central metabolic pathway genes *tktA* and *ppsA* on styrene production

To produce one molecule of l-Phe, two molecules of PEP and one molecule of E4P, which are both involved in the central metabolic pathways, are required. PEP is predominantly utilized in the phosphotransferase system (PTS), which is responsible for the translocation and phosphorylation of glucose, converting one PEP molecule to pyruvate (Fig. 1) [33]. Enhancing the expression level of PEP synthase (encoded by *ppsA*), which recycles pyruvate generated by PTS-mediated glucose transport to PEP, is an important approach for increasing the carbon flux from PEP to the aromatic amino acids pathway [33]. E4P can be directly produced by transketolase (encoded by *tktA*) or transaldolase (encoded by *talB*), and *tktA* have been demonstrated to be more effective in directing the carbon flux to the aromatic pathway than *talB* [34].

In our study, to investigate the effect of overexpression of *ppsA* and *tktA* on styrene production, styrene production of engineered *E. coli* strains harboring different constructs in shake-flask fermentation was investigated. As shown in Fig. 3b, *E. coli* BL0801 (harboring pTrc-*AtPAL2*-*FDC1*-*ppsA*-*tktA* and pACYC-*aroF*-*pheA*) accumulated 275 mg/L styrene after 24 h of fermentation with the consumption of 6.7 g/L glucose, which represents a 131 and 268% improvement over styrene production by *E. coli* BL08 (harboring pTrc-*AtPAL2*-*FDC1* and pACYC-*aroF*-*pheA*) and *E. coli* BL05 (harboring pTrc-*AtPAL2*-*FDC1*), respectively. When compared with the previous study, which overexpressed *AtPAL2* and *FDC1* in an L-Phe overproduction strain *E. coli* NST74 (*aroH*367, *tyrR*366, *tna*-2, *lacY*5, *aroF*394(fbr), *malT*384, *pheA*101(fbr), *pheO*352, *aroG*397(fbr)) and the resulting strain was able to produce 260 mg/L styrene from 15 g/L glucose [11], it seemed that the styrene titer achieved in *E. coli* BL0801 was not improved as much as expected. Several reasons might be responsible for this result. Different genetic backgrounds between *E. coli* NST74 (K-12) and *E. coli* BL21(DE3) may result in different styrene yields. Furthermore, *aroF*^*fbr*^, *aroG*^fbr^, *tyrR*, *pheA*^fbr^, and *pheO* were overexpressed in *E. coli* NST74, while *aroF*^wt^, *pheA*^wt^, *tktA*, and *ppsA* were introduced into *E. coli* BL21(DE3). Multiple isozymes encoding genes, *aroF* and *aroG*, *pheA*, and *pheO* were co-expressed, which may significantly increase the flow of central metabolic carbon to phenylalanine biosynthesis. In the case, our engineered strain only had a slightly higher styrene production than the strain using *E. coli* NST74 as a host. Therefore, different host including *E. coli* NST74 (K-12) and more genes would be considered to increase the production of styrene in the subsequent research.

During the fermentation, the growth of engineered *E. coli* strains was very slow, with OD_600_ reaching 2.0 after 24 h of induction. Therefore, the effect of induction time on styrene production was examined. As shown in Fig. 2c, styrene production and OD_600_ did not increase along with the elongation of induction time, which could possibly be owing to the inhibition of cell growth by styrene produced by the host strains. This result reconfirmed that product toxicity is a limiting factor that must be addressed in addition to metabolic regulations.

### Styrene toxicity assay

**Fig. 4:**
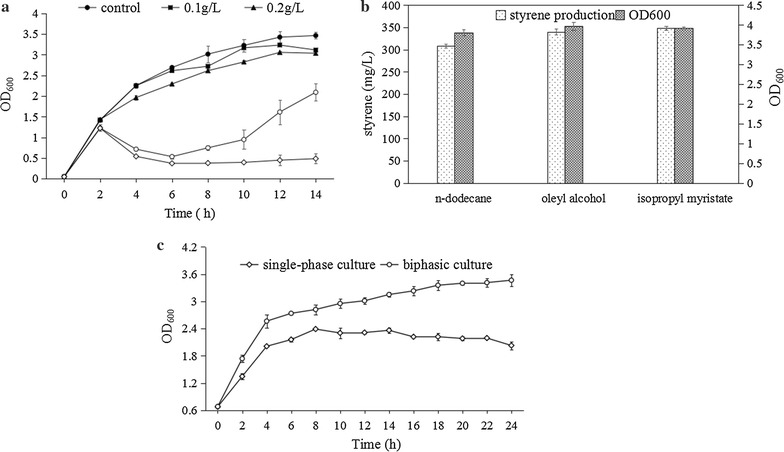
Tolerance of *E. coli* BL21(DE3) cells to styrene toxicity and ISPR with different solvents. **a** Growth response of *E. coli* BL21(DE3) cells to 0, 100, 200, 300, and 400 mg/L styrene in LB medium. **b** Effect of different solvents on styrene production and cell growth. **c** Time course of biomass by *E. coli* BL0801 cells in biphasic and single-phase cultures. Error bars represent one standard deviation from triplicate experiments

To evaluate styrene toxicity on *E. coli* BL21(DE3), the effect of exogenous addition of styrene at different concentrations (100, 200, 300, and 400 mg/L) on growing cultures was investigated. As shown in Fig. 4a, an OD_600_ of 3.4 was reached in the absence of styrene. When the concentration of styrene was less than 300 mg/L, no significant growth inhibition was observed. However, when styrene concentration was increased to more than 300 mg/L, a remarkable cell growth inhibitory effect to cell growth was detected at 0–10 h. Interestingly, after 10 h, the values of OD_600_ started to increase, which could possibly be due to the adaptation of cells to cultivation conditions with styrene or evaporation of styrene due to the insolubility of styrene in water. These findings are consistent with the previously reported styrene toxicity thresholds for *E*. *coli* NST74 [11].

After systematic optimization, the styrene output achieved 275 mg/L in the present study, which was close to the inhibitory threshold (300 mg/L). However, for economically viable and sustainable production of microbial-derived renewable styrene, the styrene titers and productivity must be ultimately improved, in other words, styrene toxicity must be overcome or effectively circumvented [11]. The accumulation of de novo synthesized biofuels or other solvent-like compounds within the cytoplasmic membrane has been shown to disrupt membrane integrity, resulting in the leakage of ions, metabolites, lipids, and proteins, as well as affecting the cells ability to maintain its internal pH and an appropriate trans-membrane proton gradient [35, 36]. Efforts have been made to improve host strains for better solvent tolerance, including introduction of efflux pumps or transporters, heat shock proteins, membrane modifications, genome engineering, random mutation, adaptive evolution, and approaches that integrated multiple-tolerance strategies [24]. In addition, ISPR and medium supplements can help to ease the burden of end-product toxicity and may be used in combination with genetic approaches. Therefore, in the present study, ISPR was used in combination with genetic approaches to increase styrene tolerance and production capacity of the host strains.

### In situ product removal (ISPR) and solvent selection

For successful application of this approach, the selection of a suitable solvent is important. Ideal solvents should be biocompatible yet non-bioavailable and display high equilibrium partitioning of the target compound over water [37]. Various kinds of solvents, such as oleic acid, oleyl alcohol, miglyol, isopropyl myristate, and polypropylene glycol have been tested for their ability to improve the production of aromatic compounds [23–25, 38]. In our study, the effect of oleyl alcohol, *n*-dodecane, and isopropyl myristate on cell growth and styrene production of the engineered strain *E. coli* BL0801 was investigated, with no-solvent culture as a control. After 48 h of shake-flask fermentation, in the presence of *n*-dodecane, oleyl alcohol, and isopropyl myristate, the styrene titers reached 304, 340, and 350 mg/L, representing 110, 124, 125% improvements, respectively, when compared with that achieved in single-phase cultures (275 mg/L) (Fig. 4b). In addition, the cell growth curves of the *E. coli* BL0801 in biphasic and in single-phase cultures were also examined. The results revealed that the cell growth was significantly increased in biphasic culture when compared with that in single-phase culture (Fig. 4c), it is presumed that isopropyl myristate could selectively remove the styrene from the reaction system, thereby maintaining the styrene concentration around the cells below the inhibitory threshold, and allowing the strains to continue styrene production, resulting in higher biphasic culture than that in single-phase culture.

Furthermore, maximum theoretical yield coefficients ^max^*Y*_*Phe*_*/Glc* were calculated from the known stoichiometry of l-Phe biosynthesis from glucose, in an engineered strain where either the PTS was inactive or PYR was being recycled back to PEP, and the maximum theoretical yield(^eng. max^*Y*_*Phe*_*/Glc*) was 0.55 g/g [20, 39, 40]. Moreover, based on the hypothesis that complete conversion of all endogenously produced l-Phe to styrene is possible (e.g., if the pathway engineered in the present study could achieve a particularly high flux), the maximum theoretical yield (^eng. max^*Y*_*styrene*_*/Glc*) was calculated to be 0.35 g/g. According to this value, in single-phase culture, *E. coli* BL0801 strains reached yields 0.041 g/g, corresponding to 12% of the ^eng. max^*Y*_*styrene*_*/Glc*, while in biphasic culture, *E. coli* BL0801 strains reached yields 0.048 g/g, corresponding to 14% of the ^eng. max^*Y*_*styrene*_*/Glc*. The above data indicate that it is possible to achieve higher yield efficiency. Major challenges may come from low enzymatic activity and flux imbalance.

In the present study, methods to improve activity of PAL, which is a rate-limiting enzyme in the styrene biosynthesis, were first considered. Significant achievements were made by enzyme engineering, such as screening of enzymes with high activity and specificity [11, 41, 42], mutating the coding sequence in the regulatory domain [12, 43, 44], and family shuffling recombines natural proteins with high sequence identity [45–47]. Currently, these strategies have not yet been applied to attain PAL with higher activity. Feedback inhibition is one of the fundamental mechanisms that regulates the synthesis of amino acids and avoids their excessive accumulation which may cause imbalanced metabolism [48, 49]. To improve the metabolic flux towards l-Phe biosynthesis, overexpression of feedback-resistant *pheA* (*pheA*^fbr^) and *aroF* (*aroF*^fbr^) genes may be effective strategies. However, mutant enzymes may decrease thermostability and catalytic efficiency. For example, the use of the *aroF*^wt^ produced much more l-Phe than *aroF*^fbr^ (Asn8-Lys) due to the decreasing thermostability of *aroF*^fbr^ [50, 51].

To overcome flux imbalances, rational strategies to regulate gene expression were developed, such as application of inducible promoters, use of non-native RNA polymerase [52], the replacement of the ribosome binding site [53], as well as multivariate modular metabolic engineering [54]. Recently, biosensors have been employed to regulate metabolic flux. Biosensor application is an effective strategy that could dynamically detect pathway flux or the levels of pathway intermediates or products and regulators that respond to sensor input and accordingly regulate enzyme expression [55]. In a report, transcription factor (TF)-based sensor, a mutated transcriptional activator *NahR* from *Pseudomonas putida*, was used to detect benzoate and 2-hydroxybenzoate accumulation in *E. coli* [56]. In another report, researchers utilized a lysine riboswitch (RNA-based) to regulate the expression of citrate synthase and control the metabolic flux of the tricarboxylic acid cycle in a lysine-producing strain *Corynebacterium glutamicum* LP917, which increased the lysine production by 63% [57]. However, most applications have been limited to natural sensor-regulators [55]. Modular scaffold strategies are also effective approaches to improve metabolic flux. A scaffold protein carrying multiple protein–protein interaction domains is used to co-localize sequential pathway enzymes that have been tagged with peptide ligands specific for the domains on the scaffold [58]. The combined use of these multi-functional enzymes might increase the yield and titer of aromatic compounds from glucose. However, a major drawback of this method is that it often results in decreased activity of one enzyme or both the enzymes [59].

Based on the above reason, our subsequent work would focus on finding feedback-resistant *pheA* (*pheA*^fbr^) and *aroF* (*aroF*^fbr^) genes with improving thermostability and catalytic efficiency. Furthermore, screening an efficient method to overcome flux imbalances would lay the foundation for industrialized production of styrene.

## Conclusion

In this study, a series of rational and systematic optimizations of styrene biosynthesis was described (Table 1). The *PAL* gene sequences from *A. thaliana*, *P. crispum*, *F. tataricum*, and *A. annua* were collected, optimized, and synthesized; these genes were based on the preferred codon usage of *E. coli*. The engineered strain *E. coli* BL01 produced 55 mg/L styrene from glucose in shake-flask cultures. Subsequently, the plasmids were optimized by evaluating the effects of copy numbers and promoters on styrene production. *E. coli* BL05 harboring plasmid pTrc-*AtPAL2*-*FDC1* strain produced 103 mg/L styrene in shake-flask fermentation, which represented a 187% increase in styrene production, when compared with that produced by *E. coli* BL01 harboring plasmid pACYC-*AtPAL2*-*FDC1*. Then, the upstream shikimate pathways genes *aroF* and *pheA* were overexpressed in *E. coli* BL0501 strain, which led to a final styrene production at titers of 210 mg/L in shake-flask fermentation, which represents a 203% improvement in styrene production, when compared with that achieved in *E. coli* BL05 strain. Moreover, when combined with the overexpression of central metabolic pathway genes *tktA* and *ppsA*, the engineered *E. coli* BL0801 produced 275 mg/L styrene. Fermentation time optimization and toxicity assays confirmed that the endogenously synthesized styrene had inhibitory effects on cell growth. To alleviate the toxic effects of styrene on the host cells, ISPR was applied. When isopropyl myristate was used as the solvent, the styrene titers reached 350 mg/L after 48 h of shake-flask fermentation, representing 127 and 636% improvements in styrene production, when compared with that achieved in cultures without solvent (275 mg/L) and the original *E. coli* BL01, respectively. To the best of our knowledge, this is the highest styrene titer produced by de novo production of styrene in *E. coli* in shake-flask cultures.

**Table 1:** Results of the systematic optimization of styrene biosynthesis in *E. coli* BL21(DE3)

Step	Optimization strategy	Strain	Plasmid	Yield (mg/L)	Improvement over previous step (%)	Improvement over original step (%)
1	Enzyme screening and codon optimization	*E. coli* BL01	pACYC-*AtPAL*-*FDC1*	55	–	–
2	Plasmid optimization	*E. coli* BL05	pTrc-*AtPAL*-*FDC*1	103	187	187
3	Co-expression of *aroF* and *pheA*	*E. coli* BL0501	pTrc-*AtPAL*-*FDC1* and pACYC-*aroF*-*pheA*	210	203	382
4	Co-expression of *aroF*, *pheA*, *ppsA*, and *tktA*	*E. coli* BL0801	pTrc-*AtPAL*-*FDC1*-*ppsA*-*tktA* and pACYC-*aroF*-*pheA*	275	131	500
5	Expression time optimization	*E. coli* BL0801	pTrc-*AtPAL*-*FDC1*-*ppsA*-*tktA* and pACYC-*aroF*-*pheA*	275	100	500
6	In situ extraction	*E. coli* BL0801	pTrc-*AtPAL*-*FDC1*-*ppsA*-*tktA* and pACYC-*aroF*-*pheA*-*tktA*-*ppsA*	350	127	636

## Methods

### Strains and media

All the strains and plasmids used in this study are listed in Table 2. *E. coli* DH5ɑ was used as the host for DNA manipulation and *E. coli* BL21(DE3) was used as the host to express protein and produce styrene. The strains were grown routinely in Luria–Bertani (LB) broth (supplemented with antibiotics if necessary). For evaluating styrene production in shake-flask fermentation, the strains were grown in a modified M9 medium consisting of the following: Na_2_HPO_4_, 6 g/L; KH_2_PO_4_ 3 g/L; NaCl, 0.5 g/L; NH_4_Cl, 1 g/L; MgSO_4_·7H_2_O, 0.492 g/L; CaCl_2_, 0.11098 g/L; Thiamine-HCl, 0.01 g/L; glucose, 15 g/L and 1 mL/L trace element solution which includes 0.37 g/L (NH_4_)_6_Mo_7_O_24_·4H_2_O, 0.29 g/L ZnSO_4_·7H_2_O, 2.47 g/L H_3_BO_4_, 0.25 g/L CuSO_4_·5H_2_O, and 1.58 g/L MnCl_2_·4H_2_O. DNA polymerase and DNA marker were purchased from TransGen Biotech. Restrictions enzymes and the DNA ligase were purchased from Thermo Fisher Scientific.

**Table 2:** Plasmids and strains used in this study

Name	Relevant characteristics	References
Plasmids		
pACYCDuet-1	P15A origin; Cm^R^; P_T7_	Novagen
pACYCDuet-*AtPAL*-*FDC1*	P15A origin; Cm^R^; P_T7_-*AtPAL*-*FDC1*	This work
pACYCDuet-*AaPAL*-*FDC1*	P15A origin; Cm^R^; P_T7_-*AaPAL*-*FDC1*	This work
pACYCDuet-*FtPAL*-*FDC1*	P15A origin; Cm^R^; P_T7_-F*tPAL*-*FDC1*	This work
pACYCDuet-*PcPAL*-*FDC1*	P15A origin; Cm^R^; P_T7_-*PcPAL*-*FDC1*	This work
pACYCDuet-*aroF*-*pheA*	P15A origin; Cm^R^; P_T7_-*aroF*-*pheA*	This work
pTrcHis2B	pBR322 origin; Amp^R^; P_Trc_	Novagen
pTrcHis2B-*AtPAL*-*FDC1*	pBR322 origin; Amp^R^; P_Trc_-*AtPAL*-*FDC1*	This work
pTrcHis2B-*AtPAL*-*FDC1*-*ppsA*-*tktA*	pBR322 origin; Amp^R^; P_Trc_-*AtPAL*-*FDC1*-*ppsA*-*tktA*	This work
pET-28a	f1 origin; Kan^R^; P_T7_	Novagen
pET-28a-*AtPAL*-*FDC1*	f1 origin; Kan^R^; P_T7_-*AtPAL*-*FDC1*	This work
pColiDuet-1	ColA origin; Kan^R^; P_T7_-*AtPAL*-*FDC1*	Novagen
pColiDuet-*AtPAL*-*FDC1*	ColA origin; Kan^R^; P_T7_	This work
Strains		
*E. coli* BL21(DE3)	*E. coli B dcm ompT hsdS*(rB^−^ mB^−^) *gal*	Takara
*E. coli* DH5α	*deoR*, recA1, endA1, hsdR17(rk^−^, mk^+^), phoA, supE44, λ^−^, thi^−1^, gyrA96, relA1	Invitrogen
BL01	*E. coli* BL21(DE3)-harboring pACYCDuet-*AtPAL*-*FDC1*	This work
BL02	*E. coli* BL21(DE3)-harboring pACYCDuet-*AaPAL*-*FDC1*	This work
BL03	*E. coli* BL21(DE3)-harboring pACYCDuet-*FtPAL*-*FDC1*	This work
BL04	*E. coli* BL21(DE3)-harboring pACYCDuet-*PcPAL*-*FDC1*	This work
BL05	*E. coli* BL21(DE3)-harboring pTrcHis2B-*AtPAL*-*FDC1*	This work
BL06	*E. coli* BL21(DE3)-harboring pET-28a-*AtPAL*-*FDC1*	This work
BL07	*E. coli* BL21(DE3)-harboring pColADuet-*AtPAL*-*FDC1*	This work
BL0500	*E. coli* BL21(DE3)-harboring pTrcHis2B-*AtPAL*-*FDC1* and pACYCDuet-1	This work
BL0501	*E. coli* BL21(DE3)-harboring pTrcHis2B-*AtPAL*-*FDC1* and pACYCDuet-*aroF*-*pheA*	This work
BL08	*E. coli* BL21(DE3)-harboring pTrcHis2B-*AtPAL*-*FDC1* -*ppsA*-*tktA*	This work
BL0801	*E. coli* BL21(DE3)-harboring pTrcHis2B-*AtPAL*-*FDC1*-*ppsA*-*tktA* and pACYCDuet-*aroF*-*pheA*	This work

### Construction of plasmids

All the primers used in this study are listed in Table 3. The primers and optimized genes were synthesized by BGI (Beijing, China). Candidate encoding genes from *A*. *thaliana* (Accession No. AEE79055.1), *F*. *tataricum* (Accession No. GQ285125.1), *P. crispum* (Accession No. CAA68938.1), and *A*. *annua* (Accession No. AKP55356.1) were optimized and synthesized with pUC57-simple as vector. The *FDC1* (Accession No. DAA12368.1) from *S. cerevisiae* encoding tCA decarboxylase was also optimized and cloned into vector pACYCDuet-1 between *Nde* I and *Bgl* II to generate pACYC-*FDC1*. The candidate *PAL* gene fragments were digested with *Nco* I and *Not* I, and cloned into the corresponding sites of pACYC-*FDC1* to create pACYC-*AtPAL2*-*FDC1*, pACYC-*AaPAL*-*FDC1*, pACYC-*FtPAL*-*FDC1*, and pACYC-*PcPAL*-*FDC1*, respectively. Then, the *AtPAL2*-*FDC1* gene fragment was digested with *Nco* I and *Xho* I, and cloned into the corresponding sites of pTrcHis2B, pET-28a, and pColADuet-1 to create pTrc-*AtPAL2*-*FDC1*, pET-*AtPAL2*-*FDC1*, and pColA-*AtPAL2*-*FDC1*, respectively (Table 2). The *E. coli* BL21(DE3) genomic DNA was used as the template for PCR amplification. The *aroF* and *pheA* genes were cloned into pACYCDuet-1 to create pACYC-*aroF*-*pheA*. The *tktA* and *ppsA* genes were cloned into pTrc-*AtPAL2*-*FDC1* to generate pTrc-*AtPAL2*-*FDC1*-*ppsA*-*tktA* using NEBuilder HiFi DNA Assembly (New England Biolabs).

**Table 3:** Primers used in this study

Primers	Nucleotide sequence^a^	Description
aroF-F	GCggatccTATGCAAAAAGACGCGCTGAAT	Start of aroF; forward primer
aroF-R	ATAAGAATgcggccgcTTAAGCCACGCGAGCCGT	End of aroF; reverse primer
pheA-F	GAagatctcATGACATCGGAAAACCCGTTAC	Start of pheA; forward primer
pheA-R	CCGctcgagTTAGGTTGGATCAACAGGCAC	End of pheA; reverse primer
ppsA-F	CGATCGCTGACGTCGGTACCctcgagTTAAGGAGGTATATATTAATGTCCAACAATGGCTCGT	Start of ppsA; forward primer
ppsA-R	CTTTACGTGAGGACATTAATATATACCTCCTTAAaagcttTTATTTCTTCAGTTCAGCCAGG	End of ppsA; reverse primer
tktA-F	TGGCTGAACTGAAGAAATAAaagcttTTAAGGAGGTATATATTAATGTCCTCACGTAAAGAGCTT	Start of tktA; forward primer
tktA-R	CTTCTGAGATGAGTTTTTGTtctagaTTACAGCAGTTCTTTTGCTTT	End of tktA; reverse primer

^a^Restriction sites are marked in lower case letters. RBS are underline

### Cultivation conditions

Shake-flask fermentation: Seed culture was prepared by cultivating the strain in 5 mL of LB medium with appropriate antibiotics at 37 °C and 250 rpm overnight. Then, 1 mL of the seed culture was then transferred into 600-mL salt water bottle containing 100 mL of fermentation medium at 37 °C and 200 rpm. When the cell density reached OD_600_ of 0.6–0.8, the cultures were induced with 0.4 mM of isopropyl-β-d-thiogalactopyranoside (IPTG), with plug added onto the bottle, and then incubated at 30 °C for an additional 48 h.

In situ extraction of styrene in shake flask: A total of 500 μL of the seed culture were inoculated into a 250 mL shake flask containing 50 mL of M9 medium and incubated aerobically at 37 °C and 200 rpm. When the cell density reached an OD_600_ of 0.6–0.8, 0.4 mM IPTG and 10 mL of desired solvent were added to the cultures, and then incubated at 30 °C for an additional 48 h.

### Styrene toxicity assay

A total of 1 mL of the seed culture was transferred into 600 mL salt water bottle containing 100 mL of fermentation medium containing styrene with different concentrations of styrene (0, 100, 200, 300, and 400 mg/L, respectively) and cultivated at 30 °C and 200 rpm. The cell growth was determined by OD_600_ measurements using a UV/Vis spectrophotometer.

### Evaluation of styrene production in engineered *E. coli* strains

After fermentation, the cultures were centrifuged at 12,000 rpm for 1 min, and 50 mL of the supernatant was extracted with 10 mL of ethyl acetate. Then, the extract was filtrated with 0.22 μm nylon membranes and analyzed by GC–MS to confirm the biosynthesis of styrene. M9 medium without inoculation and treated with the same procedures was used as a control.

GC-MS analysis was performed using Agilent 5975C system [7]. The following conditions were used: 50 °C for 1 min, which was increased at 20 °C/min to 240 °C and held for 10 min. Peak identification was based on the relative retention time and total ion mass spectral comparison with the external standard. Styrene production was quantified by GC, with following program: 50 °C for 1 min, which was increased at 20 °C/min to 240 °C and held for 1 min.

## Additional file

**Additional file 1: Figure S1.** Validation of styrene biosynthesis by engineered *E. coli*. **a** Engineered *E. coli* was cultured in LB medium and detected with GC; **b** engineered *E. coli* was cultured in M9 medium and detected with GC–MS.

## Abbreviations

*A. thaliana*: *Arabidopsis thaliana*
*F. tataricum*: *Fagopyrum tataricum*
*P. crispum*: *Petroselinum crispum*
*A. annua*: *Artemisia annua*
ISPR: in situ product removal
tCA: trans-cinnamic acid
l-Phe: l-phenylalanine
FDC1: ferulic acid decarboxylase
PAL: phenylalanine ammonia lyase
DAHPS: 3-deoxy-d-arabino-heptulosonate-7-phosphate synthetase
CM–PDT: chorismate mutase and prephenate dehydratase
GC–MS: gas chromatography–mass spectrometry
PTS: phosphotransferase system
PEP: phosphoenolpyruvate
E4P: erythrose 4-phosphate
DAHP: 3-deoxy-d-arabino-heptulosonate-7-phosphate
