# Supplementary figures and images for: A systematic optimization of styrene biosynthesis in Escherichia coli BL21(DE3)
Source: Biotechnol Biofuels. 2018 Jan 25;11:14. doi: 10.1186/s13068-018-1017-z (PMC5784704; doi:10.1186/s13068-018-1017-z)

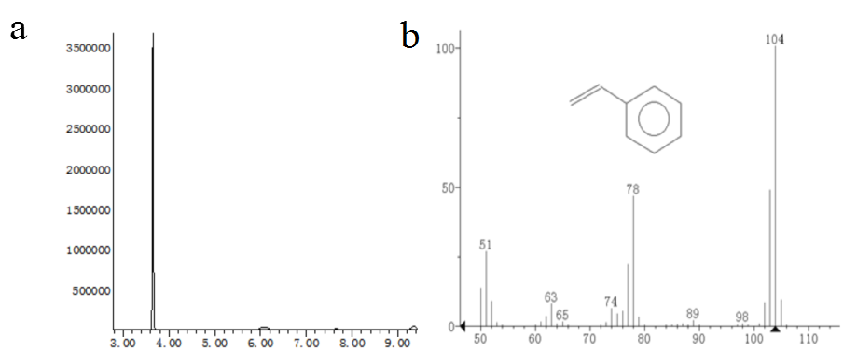

Supplement: Supplementary file 1 — Additional file 1: Figure S1. Validation of styrene biosynthesis by engineered E. coli. a Engineered E. coli was cultured in LB medium and detected with GC; b engineered E. coli was cultured in M9 medium and detected with GC–MS. [file 13068_2018_1017_MOESM1_ESM.png]
